# Quantification of Coronary Flow Reserve with CZT Gamma Camera in the Evaluation of Multivessel Coronary Disease

**DOI:** 10.5935/abc.20180196

**Published:** 2018-10

**Authors:** Ana Carolina do Amaral Henrique de Souza, Bernardo Kremer Diniz Gonçalves, Angelo Tedeschi, Ronaldo de Souza Leão Lima

**Affiliations:** 1 Universidade Federal do Rio de Janeiro (UFRJ), Rio de Janeiro, RJ - Brazil; 2 Clínica de Diagnóstico por Imagem, Rio de Janeiro, RJ - Brazil

**Keywords:** Fractional Flow Reserve, Myocardial, Coronary Artery Disease, Coronary floe reserve/methods, Diagnostic Imaging, Myocardial Perfusion Imaging

## Introduction

Evaluating patients with multivessel coronary disease using myocardial perfusion scintigraphy (MPS) remains a challenge as the extent and severity of the disease can be underestimated. This phenomenon occurs in part due to balanced ischemia and inaccuracy of traditional devices to identify small changes in coronary flow in the stress phase.^1^^,^^2^ New gamma cameras with cadmium and zinc telluride (CZT) detectors that are already commercially available have shown higher temporal and spatial resolution,^3^^-^^5^ theoretically enabling dynamic acquisition of images and calculation of myocardial blood flow (MBF) and coronary flow reserve (CFR) in an absolute way.^6^^,^^7^ This tool, whose use with positron emission tomography (PET) is already well established,^8^^-^^10^ may be promising to non invasively access three-vessel obstructive coronary artery disease (CAD) using scintigraphy and its conventional radiotracers. The objective of this case report is to describe the quantification of CFR upon diagnosis of a patient with multivessel disease whose myocardial perfusion image showed a defect not compatible with coronary angiography.

### Clinical case

**Figure 1:**
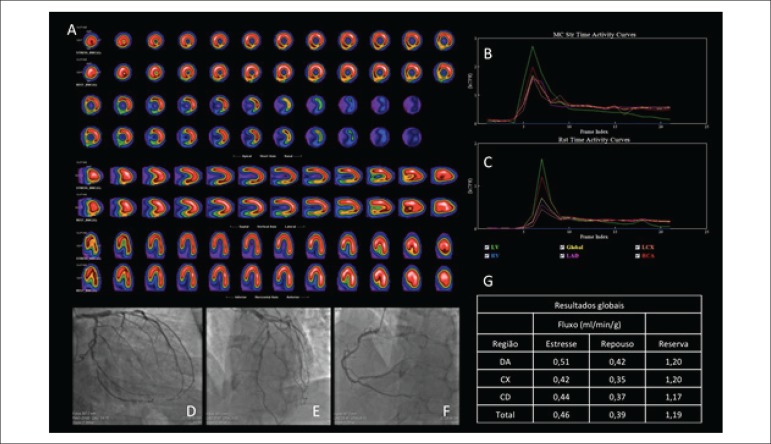
Evaluation of patient with suspected CAD. A) MPS showing small area of inferolateral ischemia. B and C) Time-activity curves in stress and rest phases, respectively, derived from the dynamic acquisition in CZT gamma camera. D, E, and F) Angiographic images showing 90% lesion in anterior descending artery, 75% lesion in circumflex artery and 50% in the middle third of right coronary artery, with 90% obstruction in posterior ventricular branch. G) Overall results of MBF quantification (in ml/min/g) and CFR in coronary territories (anterior descending artery, circumflex artery, and right coronary artery, respectively), followed by total values (last line). Reduced values of MBF and CFR are seen in all territories, which is compatible with the obstructive lesions found in coronary angiography. CAD: coronary artery disease; MPS: myocardial perfusion scintigraphy; MBF: myocardial blood flow; CFR: coronary flow reserve.

A 58-year-old patient was seen for the first time in an outpatient Cardiology clinic presenting with dyspnea on medium exertion and improvement with rest. His medical history included hypertension, dyslipidemia, and positive family history. The patient was not under regular clinical follow-up or on optimized medication. Transthoracic echocardiogram performed nine months showed no alterations and patient was referred for myocardial perfusion scintigraphy in a specialized service. A one-day protocol was performed, with rest phase followed by pharmacological stress phase using dipyridamole and ^99m^Tc-sestamibi as radiotracer at 10 and 30 mCi at rest and stress, respectively. Images were obtained in a CZT gamma camera (Discovery 530, GE Healthcare), with MBF and CFR quantified in a context of clinical research, coupled with the perfusion imaging protocol. The protocol was initiated by intravenous injection of 1 mCi of ^99m^Tc-sestamibi to place the heart within the gamma camera field of vision. The rest phase included the acquisition of dynamic images during eleven minutes, immediately followed by the perfusion images during five minutes. While the patient was still positioned in the gamma camera, pharmacological stress phase was initiated with dipyridamole (0.56 mg/kg) so that stress dynamic images could be obtained during eleven minutes and perfusion images, for three minutes. Images showed a small area of inferolateral ischemia, with no contractile alterations. Reduced CFR values were identified in all coronary territories, as well as absolute flow (ml/min/g), on rest and stress (Figure 1). After scintigraphy, symptoms persisted despite therapeutic optimization, so the patient was referred for coronary angiography, which revealed three-vessel obstructive CAD, with a 90% segmental lesion of the proximal third in anterior descending artery; 75% proximal lesion in the second diagonal branch; 75% ostial lesion in the first and third marginal branches of the circumflex; 75% segmental lesion in the posterior ventricular branch. In the right coronary artery, a long lesion of 50% was found in the middle third, in addition to a 75% lesion in the posterior descending and ventricular branches (PD/VP), with 90% impairment of the PV branch.

## Discussion

This is the first quantification report of CFR in a CZT gamma camera in our country. The protocol for image acquisition was proven safe and adequate to generate good-quality data. This case clearly represents a situation in which MPS is not able to identify the extent of ischemia due to multivessel disease. This phenomenon is in accordance with the literature, which has already described low prevalence of perfusion defects in populations of patients with three-vessel coronary obstructive disease.^1^ One of the reasons of this event is balanced ischemia. Considering that MPS only evaluates relative flow, it is based on the comparison of a myocardial wall with another whose radiotracer uptake is greater, and in situations like these an overall flow reduction occurs, generating little or no heterogeneity and, therefore, a possibly normal image.

In this context, determining myocardial flow and quantifying CFR is useful to identify high-risk patients, as they present absolute and non-relative results, like in conventional MPS. CFR can be defined as the magnitude of increased myocardial blood flow secondary to stress of any nature compared to resting flow. It is thus possible to describe not only the effects of focal epicardial obstructions, but also diffuse atherosclerosis and microvascular dysfunction, both of which are quite common in women and patients with metabolic syndrome. Previous PET studies have shown that CFR measurement can classify patients at low and high risk for cardiovascular events^9^ and therefore be used as a new tool for risk stratification.

New gamma cameras with solid and stationary CZT detectors have advantages when compared to traditional ones, with sodium-iodide detectors, as they allow for dynamic tomographic images and, theoretically, CFR quantification. Wells et al.^6^, in a pioneering work, have demonstrated a precise CFR quantification in a porcine model of resting and transitory occlusion upon stress using CZT gamma camera, paving the way for new possibilities of pilot studies with humans. Bouallègue et al.^7^ evaluated CFR in 23 patients in comparison to their angiographic data, including fractional flow reserve (FFR), and found a good correlation between CFR and the number of obstructed vessels and reduced CFR values in obstructed territories.

As seen in the present report, CFR quantification and the new methods of dynamic acquisition of myocardial blood flow constitute a current field of research that could generate knowledge about new applications of scintigraphy and bring improvements to diagnosis and management of coronary disease patients, including those with multivessel disease.
